# Mitochondrial function is enhanced by thyroid hormones during zebra finch development

**DOI:** 10.1098/rsos.240417

**Published:** 2024-07-31

**Authors:** Marlene Oefele, Michaela Hau, Suvi Ruuskanen, Stefania Casagrande

**Affiliations:** ^1^ Evolutionary Physiology Research Group, Max Planck Institute for Biological Intelligence, Eberhard-Gwinner-Strasse, Seewiesen 82319, Germany; ^2^ Department of Biology, University of Konstanz, Konstanz D-78464, Germany; ^3^ Environmental Physiology Research Group, University of Jyväskylä, Seminaarinkatu 15, University of Jyväskylä, Jyväskylä FI-40014, Finland

**Keywords:** mitochondria, thyroid hormones, cellular respiration, growth, metabolism

## Abstract

An organism’s response to its environment is largely determined by changes in the energy supplied by aerobic mitochondrial metabolism via adenosine triphosphate (ATP) production. ATP is especially important under energy-demanding conditions, such as during rapid growth. It is currently poorly understood how environmental factors influence energy metabolism and mitochondrial functioning, but recent studies suggest the role of thyroid hormones (TH). TH are key regulators of growth and metabolism and can be flexibly adjusted to environmental conditions, such as environmental temperature or food availability. To test whether TH enhancement is causally linked to mitochondrial function and growth, we provided TH orally at physiological concentrations during the main growth phase in zebra finch (*Taeniopygia guttata*) nestlings reared in a challenging environment. TH treatment accelerated maximal mitochondrial working capacity—a trait that reflects mitochondrial ATP production, without affecting growth. To our knowledge, this is the first study to characterize the regulation of mitochondria by TH during development in a semi-naturalistic context and to address implications for fitness-related traits, such as growth.

## 1. Introduction

Organisms continually adjust to their surroundings, and energy dynamics play a pivotal role in shaping these responses. Central to these dynamics is aerobic mitochondrial metabolism, which fuels cellular functions through the production of adenosine triphosphate (ATP). The significance of ATP becomes particularly evident under energy-intensive conditions, such as periods of rapid growth. However, the interplay between environmental factors, energetic needs and mitochondrial performance remains insufficiently understood. Recent research has begun to shed light on this issue, indicating that thyroid hormones (TH) may serve as a link between environmental influences and metabolic adjustment [1,2]. TH plays a central role in vertebrate development, with a significant influence on growth [1,3]. They affect cellular differentiation, proliferation [4] and metabolic regulation [5], all of which are essential for growth. At the cellular level, understanding the interaction between TH and mitochondria is crucial because their secretion is flexibly adjusted to environmental conditions, such as climatic conditions or food availability [1,2,6–8]. Aerobic mitochondrial metabolism—and hence ATP production—determines an organism’s energy metabolism [9] and thus the ability to adjust to different ecological conditions [10]. Mitochondria are known to be important drivers of life-history decisions and adjustments to ecological challenges [11]. To date, we know little about the mechanisms that mediate environmental influences on mitochondrial function, but it is plausible that they are under the influence of TH [5,12–20]. However, we are still missing ecological perspectives of this fascinating biochemical TH–mitochondria interplay.

The control of mitochondrial function, hence of energy production, is especially important during growth and development, a critical life-history stage characterized by high energetic demands. Growth requires protein synthesis and cell replication, which are processes that necessitate energy in the form of ATP. In vertebrates, optimal growth rate and large body size are related to survival [21–24] and thus ultimately determine fitness outcomes for parents and offspring. Nevertheless, only a few studies have examined mitochondria and TH in an ecological context during offspring development and most of them were carried out in birds. The development of birds, particularly during the early life period, is characterized by rapid growth and high metabolic demands [23,25]. Avian species exhibit remarkable phenotypic plasticity and adaptive strategies to cope with fluctuating environmental conditions during development, thus representing excellent models to investigate the effect of change in TH on growth and metabolism [6]. In great tit (*Parus major*) nestlings, a single TH-injection into the eggs did not affect mitochondrial respiration [26] or mitochondrial density [27] at the fledgling stage. This could mean that TH elevation during the embryonic phase does not have a lasting effect, having disappeared by the end of a hatchling’s development. However, in altricial hatchlings, TH levels have been shown to rise steadily in the first week after hatching and reach adult levels around fledging—in parallel with the hatchlings’ growth patterns, and thus, post-hatching TH and growth patterns should be studied [25]. Although some studies have addressed the effects of TH on growth in altricial nestlings, results across studies are inconclusive [25–36]. In great tits*,* prenatal TH manipulations had no effect on growth at any stage [26]. However, in collared flycatchers (*Ficedula albicollis*), prenatal TH-elevations lead to larger chicks and earlier hatching, but no long-term consequences in the nestling stage were observed [35]. Nevertheless, the environmental factors that are known to promote hormonal flexibility are manifold and it is still unknown whether and how TH fluctuations during the fledging stage affect mitochondria and growth. The effects of TH in the fledging state have mostly been studied in poultry but at concentrations exceeding physiological ranges. These studies show that both hypo- and hyper-thyroidal conditions reduce growth and development [6,37–39]. Furthermore, in red-winged blackbird (*Agelaius phoenicius*) nestlings, hyper- and hypo-thyroidal conditions led to reduced skeletal growth, compared with unmanipulated nestlings [33] and in the zebra finch (*Taeniopygia guttata*), TH-inhibition was related to decreased growth [34]. However, it has remained unclear whether these relationships are induced by pharmacological TH concentrations or in fact do occur at physiological TH concentrations as well.

To study environmentally induced TH responses, it is crucial to operate within the physiological range of TH levels, which represent the evolved levels in response to environmental signals. These studies are vital for understanding the effects of environmental challenges on life-history outcomes. For example, it is still not clear how an organism’s metabolism is adjusted to changing environmental conditions, like rising temperatures or a lack of nutrient availability—both of which have been shown to influence TH-secretion [1,2,6,7,40]—and what the consequences are for fitness and life-history outcomes. Growth is a valuable trait to study because growth patterns in early life can determine survival, especially in young passerines [41].

Furthermore, it is important to analyse the metabolic consequences mediated by TH, because TH interact with metabolism, and growth and metabolism *per se* are highly interconnected. For instance, TH influence growth hormone secretion, a core component of growth regulation [42]. Growth hormone can boost mitochondrial efficiency, metabolic rate and biogenesis in tandem with insulin-like growth factor to meet growth’s metabolic demands [43]. Thus, the effects of TH on mitochondria could be indirect, for instance via growth hormone. However, TH has also been shown to affect mitochondrial function independently of growth in multiple ways [44]. For example, by promoting the transcription of mitochondrial enzymes such as cytochrome a and c [15,19,45], via the transcription of nuclear genes, by interacting with TH-binding sites in the DNA, via cellular signalling cascades [15,46] or direct stimulation of components of the mitochondrial electron transport chain, such as complex V [15,19]. Also, TH has been shown to stimulate mitochondrial metabolic function within minutes through mitochondrial T3 binding sites or by inducing the transcription of mitochondrial genes [46]. This probably enables the organism to react to sudden changes in energetic demands. An example of a short-term adjustment like this would be the need to boost thermoregulation when experiencing a rapid drop in environmental temperature. Mitochondrial thermogenesis involves leak respiration, where energy is transformed into heat instead of ATP, and TH interacts with mitochondrial uncoupling proteins [19]—the primary drivers of leak [47,48]. While TH is involved in rapid mitochondrial adjustments such as for thermoregulation, another important function of TH is the adjustment of mitochondrial function to sustained physiological demands, such as growth or long-lasting environmental challenges [19]. These adjustments can be conveyed through epigenetic programming by the modification of methylation patterns during development [49,50] or through promoting the transcription of mitochondrial genes [46].

Here, we experimentally tested the hypothesis that TH plays a role in stimulating mitochondrial function during development, in addition to augmenting growth. To be able to transfer our findings to natural systems, we repeatedly elevated circulating levels of T3 (triiodothyronine, the biologically active thyroid hormone) within physiological ranges during the most rapid post-hatch growth period in captive zebra finches to address the following questions: (i) do elevated TH concentrations enhance growth in zebra finch nestlings? We expected TH-treated nestlings to have faster growth rates in both skeletal size and body mass. As a result of accelerated growth, TH-treated chicks may either have larger body sizes at the time of fledging than control chicks, or alternatively, reach the same final size as control chicks but at an earlier age. (ii) Is mitochondrial function enhanced by sustained elevated TH levels during growth? We expected TH to accelerate mitochondrial respiration rate and oxidative phosphorylation, which are the primary determinants of ATP production rate.

## 2. Methods

### 2.1. Model species and housing

Zebra finches from a local captive Seewiesen population were allowed to breed for 23 weeks in two communal indoor aviaries. The local captive zebra finch population was acquired in 2002 (231 individuals at the time) from a colony held since 1985 by T. R. Birkhead at Sheffield University, UK. For more information, see population no. 18 in [51]. Each of the aviaries was equipped with 30 wooden nest boxes and 7−11 open nesting cups, maintained at a constant temperature of 21°C with humidity at 59%, at a light : dark photoperiod of 14 : 10 h. The birds were provided with white cotton and coconut fibres as nesting material, commercial seed mixture, supplementary commercial egg food, cuttlefish bone, grit and water ad libitum. The birds were housed in communal aviaries where access to seeds and egg food was constrained by offering a 2 : 1 food-to-husk ratio to mimic naturally challenging food resources [52]. Once per week, the diet was supplemented with salad and multivitamins. The husk–seed mixture was provided in open boxes (60 × 40 × 12 cm) and the husk–egg food mixture in flat plates. Food dishes were placed on the floor. Nests were checked once per day for new hatchlings, and new hatchlings were colour marked with food colouring on their head or back down feathers. As part of a separate study, at 1 or 2 days post-hatch (dph), each chick was cross-fostered to create either large (six or seven chicks) or small broods (two or three chicks). All chicks included in this study were reared in enlarged broods and thus experienced a challenging environment, which included a possibility of food shortage, the need for enhanced begging rates and competition for space [53]. The mean brood size during the experiment for the total duration was 5.56, s.d.: 0.699. This number is slightly lower than the initial brood size of six or seven chicks, owing to naturally occurring death of chicks throughout the nestling period and asynchronous fledging.

### 2.2. Study design and TH administration

**Figure 1.**
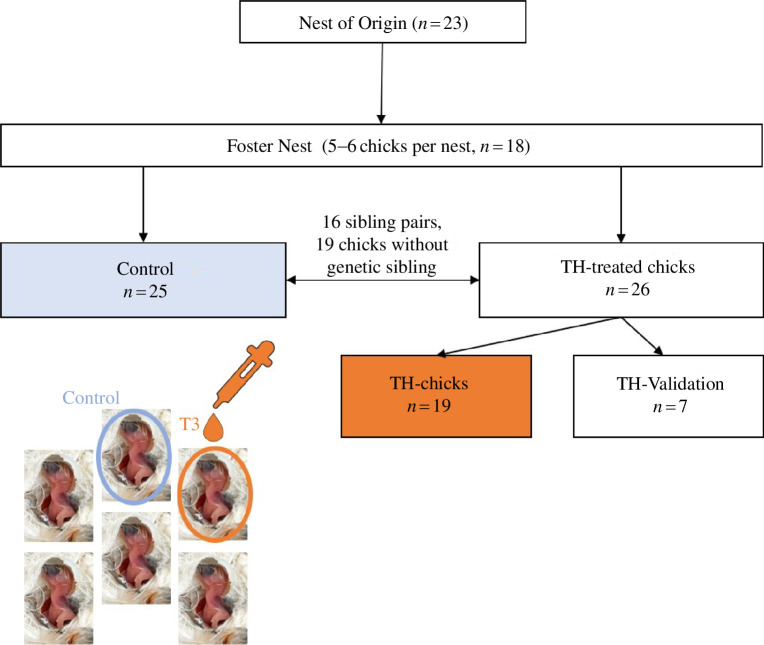
Schematic study design and sample sizes per group. The assignment of chicks to control (blue square), TH-chicks (experimental group, orange square) and TH-validation (not included in analysis) groups is shown, as well as an example of the within-brood design in the foster nests. Photo credit: Kim Holzmann.

The thyroid gland produces mainly thyroxine (T4), which is converted to triiodothyronine (T3) by deiodinase enzymes [54] while maintaining a certain T3/T4 ratio in the plasma. Our treatment consisted of T3, as it is the biologically active form of TH, because of its generally higher receptor affinity than T4 [55]. We aimed to elevate T3 within physiological ranges during the main growth phase; however, the daily T3 production rate of hatchling zebra finches is not known. Thus, we considered the daily secretion rate of 1–3 μg T4 per 100 g body mass as shown in other bird species [56] in combination with data on the T3 to T4 ratio, which is around 0.5, as shown in the circulation of developing zebra finches [57]. We selected a treatment dosage of 0.5 μg T3 per 100 g body mass. We achieved this dosage by multiplying the lower range of the known secretion rate of 1–3 μg T4 per 100 g body mass with the known T3/T4 ratio: (1 μg T4 per 100 g body mass) × (0.5 T3/T4 ratio). We decided to use the lower range of the known T4 secretion rate for our calculation to avoid overdosing. All control chicks received the vector solution. Since mitochondrial function is sensitive to stress, for example, mediated via glucocorticoids [58], our aim was to minimize handling stress as much as possible. For this reason, T3 was administered every second day (i.e. on dph 5, 7, 9, 11 and 13 with a 200 μl pipette, adjusted to the exact body mass of each chick, ± 0.01 g) to obtain peaks of elevated T3 levels throughout the main growth period between 5 and 14 dph [33,59]. Blood sampling was done at 14 dph (*ca* 20 h after T3 administration in the experimental group) to measure circulating T3 concentrations and mitochondria function. T3 was administered in a within-brood design, hence within each brood, one randomly chosen chick received T3 (*n* = 19). For the control group, either a sibling of the TH-treated chicks (hatched in the same nest of origin), was chosen to account for potential genetic similarities (sibling pairs between TH-treated and control chicks *n* = 16), or, because of a lack of availability, chosen randomly (total chicks without genetic sibling *n* = 19). For an illustration of the study design, see figure 1. Each foster nest contained a varying number of focal chicks that are included in this study: 11 nests contained one or two TH-chicks and one–three control; four nests contained one TH-chick each and no controls; one nest contained one control chick and no TH-chicks; and two nests contained three control chicks and no TH-chicks.

### 2.3. TH-validation group

A separate group called ‘TH-validation’ (*n* = 7) was used to confirm that the treatment led to the desired peaks of elevated plasma T3 shortly after each administration. In the TH-chicks, the last T3 dose was given at noon on 13 dph and blood sampling (see §2.6) was done at 14 dph at around 09.00. Hence, it is likely that T3 given on 13 dph had been metabolized in large parts before blood sampling [33,59,60], and therefore T3 plasma levels at that time point do not reflect the full extent to which T3 plasma levels where raised on multiple occasions throughout the treatment period. To confirm that the treatment did in fact raise T3 plasma levels as expected, we added the so-called ‘TH-validation’ group (*n* = 7) to our experiment. To our knowledge, the oral uptake rate of T3 in birds has not been tested while in humans T3 plasma levels reach their peak about 2.5 h after oral administration [60]. Since birds have a faster metabolism than humans—e.g. mass-specific basal metabolic rates are 30–40% higher in birds than mammals [61]—we expected plasma T3 levels to reach peak levels around 1 h post administration in our nestlings. To confirm this, the TH-validation group received their last treatment dose on 14 dph, 1 h before taking the blood samples, instead of on 13 dph, like the rest of the birds in the study. Other than the final dose lagging for 1 day (14 dph instead of 13 dph), the TH-validation group was treated in the same way as the TH-chicks (the experimental group). The TH-validation group was used to confirm the T3-treatment and included in the growth curve analysis, but was not part of any other analysis in this study.

To verify that the treatment elevated T3 plasma levels in the TH-chicks as expected, a separate regression model was run in which plasma T3-levels of the TH-validation group were included in the TH-chicks group, see §2.9. The TH-validation group was not considered for any other analysis.

### 2.4. TH and vector solution

For the preparation of the hormone solution, crystalline T3 (3,3′,5‐triiodo‐L‐thyronine, greater than 95% HPLC, CAS number 6893‐02‐3, Sigma‐Aldrich) was first dissolved in 0.1 M NaOH (NaOH reagent grade, greater than or equal to 98%, anhydrous pellets, CAS number 1310-7-3-2, diluted in MilliQ water) and then this T3–NaOH solution was diluted at 1 : 60 with tap water [62]. The concentration of the solution was adjusted, so that a 1 µl final solution could be administered per gram body mass of the chick [63], with a dosage of 0.5 µg T3 per 100 g body mass. For the vector preparation, 0.1 M NaOH solution was diluted at 1 : 60 with tap water. Prepared vector and treatment aliquots were stored at −20°C and kept on ice until use. Thawed aliquots were stored in the fridge and used within 1 day.

### 2.5. Chick growth

In order to monitor the hatchlings’ development and to adjust the treatment dosage (see §2.2), body mass (KERN, ± 0.01 or Pesola spring scale, ± 0.25 g) and tarsus length were taken at 0, 3, 5, 7, 9, 11, 13 and 14 dph. Because TH can affect either skeletal growth [33], muscular growth [64], fat deposition [65] or potentially all of these factors, we assessed body condition in addition to tarsus length and body mass measures, to test if the effects of TH on either of these growth parameters affected body condition. To assess body condition, we used the scaled mass index (SMI) [66] between body mass and tarsus length at 5, 7, 9, 11, 13 and 14 dph. Pearson’s correlation coefficient for body mass and tarsus lengths ranged from 0.76 to 0.85, with *p*-values less than 2.2 × 10^−16^. Growth rates were calculated separately for tarsus length and body mass and from here on will be called ‘skeletal growth rate’ and ‘mass growth rate’, respectively. Growth rates were calculated for 5 dph (the start of the treatment), 7, 9, 11 and 13 dph in the following way (see also [67]):


(Tarsus(t)–Tarsus(s))/(t–s),

where *s* is the preceding measurement to time point *t.*

The time point 14 dph was not included in growth rate calculations. While all other measurements were taken at noon, the measurement at 14 dph was taken in the morning shortly before blood sampling. Thus, body mass was expected to be lower at 14 dph compared with 13 dph because of the time of the day. Tarsus lengths did not change between 13 and 14 dph.

### 2.6. Blood sampling

Blood samples were taken within 6 min of entering the aviary by brachial venipuncture from individuals at 5 dph, for a separate study, and at 14 dph, to obtain plasma samples and mitochondrial measures for this study. A maximum amount of 1% of the nestling’s body mass was taken. Blood was stored immediately on ice and centrifuged within 2 h at 2000*g* for 10 min. Plasma was stored at −80°C and analysed within six months. Red blood cells (RBCs) were processed immediately for mitochondrial measurements, according to [68].

### 2.7. Mitochondrial measurement

Avian erythrocytes possess functional mitochondria [69,70], allowing us to assess mitochondrial function in intact RBCs [68]. We used intact RBCs to analyse mitochondrial traits in the natural physiological state of the bird, without artificially altering their immediate environment, e.g. substrate availability in the cell. We included the following five mitochondrial variables in this study: cellular metabolic rate (CMR)—the total amount of oxygen consumption in the current cellular condition; proton leakage (‘Leak’)—the portion of mitochondrial respiration not used for ATP production; oxidative phosphorylation (‘OxPhos’, calculated by subtracting leak respiration from CMR)—the portion of the mitochondrial O_2_ consumption currently used for ATP production; maximal working capacity of the electron transport system (‘ETS’)—the maximum capacity of the mitochondria to produce ATP under the current cellular conditions, including substrate and electron chain enzyme availability; and the flux control ratios (FCR) L/CMR (ratio between leak and CMR) [68]. For assessing mitochondrial function, a Clark electrode high-resolution respirometer (Oxygraph-2k, Oroboros Instruments, Innsbruck, Austria) was used on intact RBCs. An aliquot of 7–30 µl RBC (mean 16.33 µl; s.d.: 5.99 µl) was taken after centrifugation of the blood sample from the bottom of the tube with a cut-off pipette tip. All measurements were normalized for the volume of RBC used for the analysis. The RBC aliquot was then gently homogenized in cold respiratory MiR05 buffer (0.5  mmol l^−1^ EGTA, 3  mmol l^−1^ MgCl_2_, 60  mmol l^−1^ potassium lactobionate, 20  mmol l^−1^ taurine, 10  mmol l^−1^ KH_2_PO_4_, 20  mmol l^−1^ Hepes, 110  mmol l^−1^ sucrose, 15  mmol l^−1^ fatty acid-free bovine serum albumin, pH 7.1). The RBC-buffer solution was centrifuged at 500*g* for 5 min and the supernatant was removed. Washed RBCs were resuspended in fresh Mir05 buffer at 40°C and added to the respirometer chamber. Then, RBC oxygen consumption was quantified according to the following protocol: (i) CMR, often called ‘Routine’ respiration, is the basal respiration of the cell; (ii) 1  µg ml^−1^ oligomycin was added to the chamber to inhibit ATP-dependent oxygen consumption and to assess proton leak, here called ‘leak’ respiration; (iii) 1  µmol l^−1^, titration of carbonyl cyanide *m*-chlorophenyl hydrazine, a mitochondrial uncoupler was added, to assess the maximal capacity of the electron transport chain, so-called ‘ETS’; (iv) 5  µmol l^−1^ antimycin a, to quantify non-mitochondrial oxygen consumption. Non-mitochondrial oxygen consumption was subtracted from all measures above [68].

### 2.8. T3 extractions—liquid chromatography–mass spectrometry

In brief, 15 µl of plasma was mixed in methanol and spiked with a known amount of ^13^C_12_-T4 (Larodan, Sweden) to track recovery. We first performed liquid–liquid extraction with chloroform and methanol. Samples then went through solid phase extraction using chloride-form anion exchange resin (Bio-Rad) for purification and were dried under N_2_. The final products were re-dissolved in 0.01% NH_3_ and plasma T3 and T4 were simultaneously measured using a validated nano-flow liquid chromatography–mass spectrometry (LC-MS) protocol, for details see [71]. ^13^C_6_T3 and ^13^C_6_T4 (from Sigma) were spiked in each sample as internal standards, and T3 and T4 were quantified using peak area ratios of sample to its internal standard. Several quality control samples were also included. Each sample was analysed once, and across sample types, the inter-assay average CV% was 6.9 for T3 and 4.4% for T4 [71]. MS data were acquired automatically using Thermo Xcalibur software (Thermo Fisher Scientific) and analysed using Skyline [72].

### 2.9. Statistics

All statistical analyses were done in R, version 4.2.2 [73]. We used generalized linear models via the stan function from the package ‘rstanarm’, version 2.21.3 [74] to calculate posterior means and 95% credible intervals (CrI), from 4000 simulated values from the joint posterior distribution of the model parameters. The prior distribution of rstanarm can be classified as weakly informative, with a Gaussian distribution and scaling based on the distribution of the data. The default prior of each variable is a normal distribution centred at 0. The scale (s.d.) of this prior is calculated by the program in a data-dependent way, with the scale that is a function of the response variable (see electronic supplementary material, table S5, where priors for each dependent variable have been reported). Priors have a mean of zero for all group differences, thus avoiding bias towards signals from either the experimental or control group. The mean of the intercept is pooled from data from both groups [75]. The specific prior parameters can be found in electronic supplementary material, table S5. We modelled the response variables per predictors of interest via a Gaussian error distribution and the ‘Identity’ link function. We report statistical uncertainties of the estimates using 95% CrI and posterior probabilities of specific hypotheses for each statistical test. Further, we calculated the posterior probabilities (*P*), by assessing the proportion of simulated values in line with the hypothesis, for each model predictor [76]. Residual distribution was assessed by using normal quantile–quantile plots, Tukey–Ascombe plots and by plotting residuals against leverage. Model convergence and fit were assessed via the function ‘shinystan’ in the ‘rstanarm’ package. Furthermore, the *R*^2^ was calculated, as well as the residual variance of the model and the variance of the random factors.

To test for the effect of the T3 treatment on focal parameters, the variable ‘Treatment’ (Control: *n* = 25; TH-chicks: *n* = 19) was used as a predictor. In all models, the nest of birth ‘nest of origin’ (*n* = 23) was included as a random effect, to control for genetic similarities between siblings. To control for different rearing conditions across nests, ‘foster nest’ (*n* = 18) was also included as a random factor in all models. To account for possible time effects (23 weeks of the experiment), we included sampling order representing the overall hatching order as a covariate in all models. Sampling order is a continuous sequence of the order of birth of individuals, similar to Julian dates. All continuous predictor variables were standardized by computing *z*-scores ((*x*-mean)/s.d.). For some individuals, data were missing (e.g. low amount of blood, failed mitochondrial measurements, missing data), hence sample sizes are slightly different between the models (see electronic supplementary material, tables S2–S5). Plots were generated using the package ‘ggplot2’ [77].

In order to assess whether the T3 plasma levels differed between the groups, and whether this difference was enhanced by the TH-validation group (see §2.3), we ran one model in which the TH-validation group was included in the TH-chicks group (Control: *n* = 25; TH-chicks including TH-validation group: *n* = 26) and one model without the TH-validation group (Control: *n* = 25; TH-chicks without the TH-validation group: *n* = 19). T3 values were log-transformed to achieve a normal distribution of the data and to obtain the best fit of the models.

Frequentist results can be found in electronic supplementary material, tables S6–S8.2. If one of the random factors (1|nest of origin) or (1|foster nest) explained close to 0 variance in the frequentist models, it was removed from the model to avoid convergence issues. Results from frequentist models are the same compared with Bayesian interference across all models.

### 2.10. Morphometrics

To test the hypothesis that the TH-treatment positively affected the aforementioned morphological measures, we ran one model with tarsus length at 14 dph, one with body mass at 14 dph and one with SMI at 14 dph. We first tested for a difference in baseline measures at 5 dph in all three variables, by running one model per variable with the baseline measure at 5 dph as an outcome variable. As sampling order did not have a meaningful effect in the model for tarsus length at 5 dph, we removed it because of convergence issues. Tarsus length at 5 dph, hence before the onset of the treatment, was smaller in TH-chicks (estimate [95% CrI], *P*(*ß* < 0): −0.59 [−1.028; −0.163], 0.995. See electronic supplementary material, table S3, figure S1). There was no difference between the groups in body mass at 5 dph and SMI at 5 dph (see electronic supplementary material, table S3 and figures S2 and S3). We controlled for baseline measures at 5 dph in all three models testing the parameters at 14 dph (tarsus length, body mass, SMI), to account for the relatively high but natural variation at 5 dph, which can be seen in the dispersion of the raw data (electronic supplementary material, figures S1–S3). In this way, we could disentangle the effects of the treatment from existing pre-treatment differences.

To test for differences in growth between the groups, we used generalized additive mixed models (GAMMs) with the rstanarm function stan_gamm4 (see [67]). We included ‘treatment’ as a fixed effect and a smoothing term with an interaction between age and treatment, to allow for different shapes of the curves in the two groups. Further, we included random intercepts for ID over Age to account for differences in individual growth trajectories. GAMMs were calculated for skeletal and mass growth rates, as well as for body mass (g), tarsus (mm) and SMI for the time points 5 dph (start of the treatment), 7, 9, 11 and 13 dph.

### 2.11. Mitochondria

To test for differences in the five mitochondrial traits of interest (CMR, OxPhos, ETS, Leak and FCR L/CMR) we ran one model per trait, with the trait being the response variable. CMR, OxPhos, ETS and Leak were log-transformed to achieve a normal distribution of the data and to obtain the best fit of the models. During the treatment, we administered T3 every second day to obtain peaks of T3 plasma levels, with the aim of regularly elevating T3 throughout the main growth phase. In humans, TH have been shown to affect mitochondria differently in the short term (within minutes or hours after treatment) versus the long term (within weeks) [15]. Our study question aimed to address the effects of elevated T3 on mitochondria throughout the main growth phase. Acute levels of T3 at the time of sampling may differ between individuals, for example owing to varying speeds of metabolizing the T3 or the feeding status at the time of administration. Thus, we included acute T3 plasma levels as a covariate to control for immediate effects of T3 on mitochondrial function that may differ from the effects of prolonged elevated T3 levels throughout the treatment period. Furthermore, we included body mass at 14 dph (which was not statistically meaningfully affected by the treatment, see electronic supplementary material, table S3) as a covariate, to control for body mass differences not attributed to the treatment. We re-ran the models for mitochondrial traits without acute T3 levels to test whether the results changed, and we did not find a difference in either model fit or meaningfulness of response variables (see electronic supplementary material, table S4.1). Tarsus length before the onset of the treatment (at 5 dph) happened to be smaller in TH-chicks than in control chicks, while the groups did not differ in tarsus length at 14 dph. To confirm that mitochondrial traits were not affected by potential catch-up growth, we re-ran the models for mitochondrial traits with tarsus length at 5 dph as a covariate. Body mass at 14 dph was then removed from the models since body mass at 14 dph is expectedly highly dependent on tarsus length at 5 dph. Tarsus length at 5 dph did not have a meaningful effect on mitochondrial traits, nor did model fit or meaningfulness of outcome variables change (see electronic supplementary material, table S4.2 for full outputs of models including tarsus length at 5 dph).

## 3. Results

### 3.1. TH-treatment

**Figure 2.**
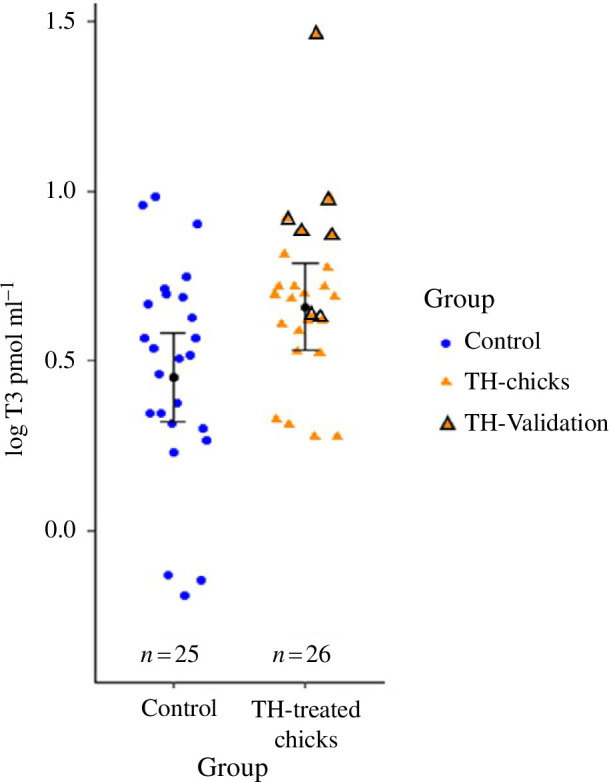
Log (T3) plasma concentrations in pmol ml^−1^ in control (blue circles) and total TH-treated chicks (orange triangles, including TH-validation and TH-chicks). The TH-validation (*n* = 7) group is marked by black lines around orange triangles. TH-chicks (*n* = 19) are shown in orange triangles and are the focal experimental group of this study. Filled black circles and error bars show the group estimated means and corresponding 95% CrIs.

As expected, T3 concentrations 1 h after administration in the separate validation group ‘TH-validation’ (pmol ml^−1^ mean: 2.568, s.e.: 0.313), and in the experimental group about 24 h after administration (pmol ml^−1^ mean: 1.825, s.e.: 0.067) were increased and within the range of the physiological levels found in the control group (pmol ml^−1^ mean: 1.68, s.e.: 0.991). T3 plasma levels in the TH-chicks ranged from 1.315 to 2.25 pmol ml^−1^, which is within the range of 0.825 to 2.674 pmol ml^−1^ of the control group. As expected, T3 levels in the TH-chicks were statistically marginally higher than in the control group (pmol ml^−1^ estimate [95% CrI], *P*(*ß* > 0): 0.127 [−0.016; 0.268], 0.962) and meaningfully higher when TH-validation chicks and TH-chicks were analysed together (pmol ml^−1^ estimate [95% CrI], *P*(*ß* > 0): 0.206 [0.066; 0.348], 0.997) (see figure 2, electronic supplementary material, table S2). Furthermore, T3-levels were similar to those observed in nestlings of other passerine species, red-winged blackbirds (*Agelaius phoenicius*) [33] and great tits [78].

### 3.2. Growth

TH-chicks had smaller tarsus lengths at 5 dph before the onset of the treatment (estimate [95% CrI], *P*(*ß* < 0): −0.59 [−1.028; −0.163], 0.995; see electronic supplementary material, table S3, figure S1). The last treatment dose was given on 13 dph and 1 day after the last treatment dose, on 14 dph, the TH-chicks did not differ in body size (in tarsus length or body mass) compared with the control group (tarsus length in mm estimate [95% CrI], *P*(*ß* < 0): −0.006 [−0.327; 0.317], 0.514). Body mass in grams estimate [95% CrI], *P*(*ß* < 0): −0.381 [−0.97; 0.21], 0.906; see electronic supplementary material, figures S4 and S5). Starting values at 5 dph were included in the models to control for differences in body size before the onset of the treatment. The treatment also did not have a strong effect on body condition, as SMIs at 14 dph were not clearly different between the groups, however, the credible interval of the estimate of the TH-chicks only marginally overlaps 0, meaning that TH-chicks did have slightly lower body condition (estimate [95% CrI], *P*(*ß* < 0): −0.537 [−1.197; 0.111], 0.945; see electronic supplementary material, figure S6). Models on SMI at 14 dph were controlled for starting values at 5 dph. For the main results, see table 1. For descriptive statistics, see electronic supplementary material, table S1.

**Table 1.** Estimates, 95% credible intervals and posterior probabilities (*ß* > 0 or *ß* < 0) for treatment effects on (1) morphometric measures and (2) mitochondria traits. Meaningful factors with credible intervals not overlapping 0 are given in bold.

	response variable	estimate	95% CrI	*P*(*ß* < 0)
morphometrics				
	tarsus 14 dph (mm)	−0.006	−0.327; 0.317	0.514
	body mass 14 dph (g)	−0.381	−0.97; 0.21	0.906
	SMI 14 dph	−0.537	−1.197; 0.111	0.945

	response variable	estimate	95% CrI	*P*(*ß* > 0)
mitochondria (pmol s^−1^ mg^−1^)				
	log CMR	0.172	−0.015; 0.354	0.965
	log OxPhos	0.167	−0.059; 0.399	0.928
	**log ETS**	**0.246**	**0.048; 0.448**	**0.993**
	log leak	0.09	−0.24; 0.428	0.709
	FCR L/CMR	−0.013	−0.111; 0.083	0.391

**Figure 3.**
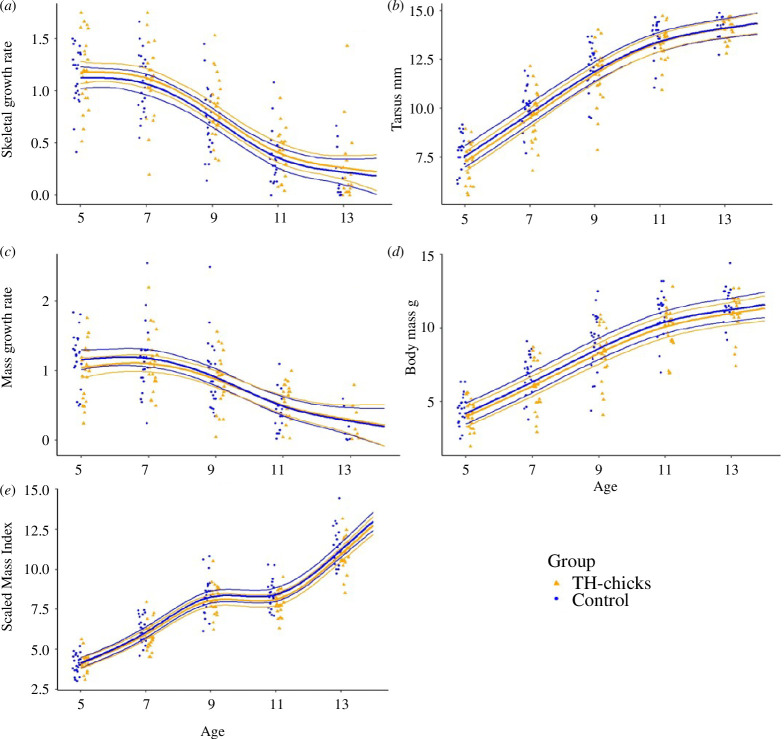
Growth curves in control (blue circles) and TH-treated chicks (orange triangles) over the treatment duration. Thick lines are regression lines estimated via GAMMs and thin lines depict 95% CrIs of (*a*) skeletal growth rate (*b*) tarsus length in mm, (*c*) mass growth rate, (*d*) body mass in g and (*e*) scaled mass index.

GAMMs were used to generate curves for skeletal and body mass growth rates, and SMI for tarsus length, for body mass or throughout the treatment duration (5–13 dph). The curves did not differ between the experimental groups, see figure 3. The interaction term between age and treatment group was not meaningful, nor were the main effects of the treatment. For full model outputs of growth, see electronic supplementary material, table S3.1.

### 3.3. Mitochondria

**Figure 4.**
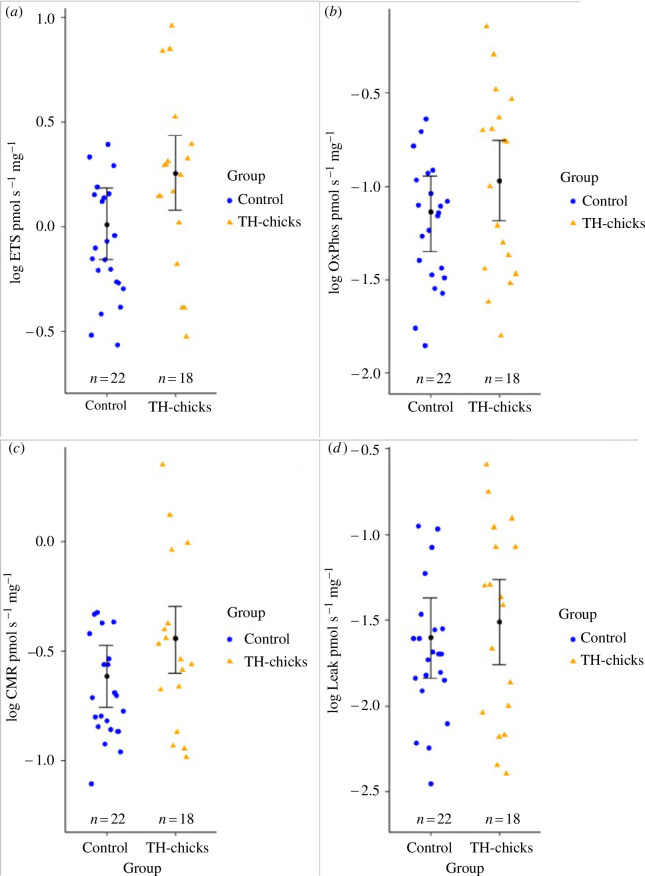
Mitochondrial traits in control (blue circles) and TH-treated chicks (orange triangles). Black circles and error bars show estimated means and 95% CrIs of log-transformed respiration rates (pmol s^−1^ mg^−1^). (*a*) Maximum electron transport chain capacity ‘ETS**’**; (*b*) oxidative phosphorylation ‘OxPhos’; (*c*) cellular metabolic rate ‘CMR’; (*d*) leak respiration ‘Leak’.

The maximum electron chain capacity (ETS) was clearly higher in TH-chicks compared with control chicks (log estimate [95% CrI], *P*(*ß* > 0): 0.246 [0.048; 0.448], 0.993; see figure 4*a*), thus elevated T3 lead to a higher capacity for ATP-production. CrIs overlap 0 for the final step of ATP-production—oxidative phosphorylation (OxPhos) (log estimate [95% CrI], *P*(*ß* > 0): 0.167 [−0.059; 0.399], 0.928; see figure 4*b*) and the overall mitochondrial oxygen consumption—cellular metabolic rate (CMR) (log estimate [95% CrI], *P*(*ß* > 0): 0.172 [−0.015; 0.354], 0.965; see figure 4*c*). These results lean towards the expected higher ATP production rate in the treatment group because the overlap with 0 is relatively small for both OxPhos and oxygen consumption (CMR). Leak respiration, see figure 4d, which is the portion of mitochondrial metabolic rate that is not dedicated towards ATP-production, and FCR L/CMR, the mitochondrial efficiency in ATP-production, clearly did not differ between treatment groups. For the main results, see table 1. For full model outputs on mitochondria, see electronic supplementary material, table S4. For descriptive statistics, see electronic supplementary material, table S1.

## 4. Discussion

Here, we tested whether TH administered within physiological levels altered mitochondrial function in developing birds during the main growth phase between hatching and fledging. Supporting our predictions, TH-chicks had enhanced mitochondrial capacity in the form of higher ETS (maximal ETS). In line with our findings, in rats, TH administration leads to enhanced transcription and synthesis of mitochondrial electron transport chain components like cytochrome a and c [45,79]. ETS can be viewed as the structural capacity of the mitochondria to produce ATP and is determined by the components of the electron transport chain. The electron transport chain of the mitochondria induces a proton gradient, which, in addition to enabling ATP synthesis, processes metabolites that are used in the tricarboxylic acid (TCA) cycle. The TCA cycle represents a separate stage of cellular respiration which produces crucial agents like nicotinamide adenine dinucleotide (NADH) and flavin adenine dinucleotide (FADH_2_) that support anabolic processes of the cell, such as the formation of amino and fatty acids. More specifically, the enzyme succinate reductase (Complex II of the electron transport chain) is part of the TCA cycle and is responsible for transforming succinate into fumarate—the step that links OxPhos with the TCA cycle [80,81]. However, contrary to OxPhos, the functioning of the electron transport chain represented by ETS does not directly depend on the availability of ADP, the substrate needed to complete the final step of ATP production in Complex V (i.e. ATP synthase) [82], which could explain why these traits were differentially influenced by the TH-treatment.

When it comes to OxPhos and CMR, CMR was marginally higher in T3-treated chicks compared with control chicks and OxPhos tended to be higher in TH-chicks. One possible explanation for why OxPhos only showed a tendency but was not clearly higher in our experimental group compared with control chicks could be an adjustment of the mitochondria to the repeated T3 exposure throughout the treatment. Several studies have shown that T3 rapidly increases OxPhos [18], and it has been shown that OxPhos activity can change flexibly depending on environmental conditions in different vertebrate species. In fish for example, OxPhos enzyme activities can vary with nutrient availability [83–85], environmental temperature [86,87] and pollutants [88,89]. However, long-term exposure to elevated TH levels may over time lead to metabolic adjustment in the usage of ATP. In our study, OxPhos may have been elevated initially at the beginning of the treatment as a response to elevated TH-levels. Nevertheless, ATP production can be limited by substrate availability, which may have not been elevated in a similar way over the course of the treatment. A similar adjustment is also plausible in CMR, to which OxPhos contributes about 70%. In adult pied flycatchers (*Ficedula hypoleuca*), for example, mitochondrial respiration rates changed with varying energetic demands during reproduction [90], which shows that CMR can be flexibly adjusted in relatively short timescales. It would also be plausible that OxPhos was not substantially affected at any time point, and merely slightly elevated because of the effect of TH on other mitochondrial parameters, for instance, the TCA cycle (see previous paragraph). We suggest future studies consider different intervals of assessing mitochondrial function and TH during the main growth phase. This could help to understand whether OxPhos is adjusted over time to increased TH levels or whether OxPhos is not directly affected by TH at any treatment duration.

Regarding growth, tarsi of experimental chicks happened to be shorter at 5 dph compared with control chicks. Chicks were cross-fostered at 1 or 2 dph, when the difference between the tarsi lengths was not evident yet. Thus, we were not able to distribute chicks to create groups with equal tarsi lengths at 5 dph. The difference in tarsi lengths between the groups had vanished by day 14, when bone development was considered complete [53]. The TH-treatment did not have an effect on growth parameters, such as growth curves for tarsus length, body mass or curves for skeletal and body mass growth rates. However, the unequal distribution in starting values of tarsus lengths before the onset of the treatment masks any clear effects of the treatment on skeletal growth. Even so, it is possible that the T3-treatment enabled compensatory [91] skeletal growth, in line with Wilson & McNabb [59]. The T3 treatment probably enabled these nestlings to catch up to the size of the control group by 14 dph and thus probably boosted their survival chances. Since growth and metabolism are highly interconnected, it would be plausible that catch-up growth in the TH-chicks would affect mitochondrial function regardless of the TH-treatment, to support the increased metabolic demands of catch-up growth. However, when accounting for tarsus length at 5 dph in the mitochondrial models, we did not see an effect of tarsus length at 5 dph, nor did the results for mitochondria change, suggesting that any potential catch-up growth effects did not affect mitochondrial function. Nevertheless, TH could have promoted growth hormone (GH) secretion [92,93], which is particularly active between 5 and 14 dph. GH could then boost OxPhos and CMR to meet the high energy demands of this rapid developmental phase, which may have waned by 14 dph when most growth has occurred. Elevated ETS at 14 dph could reflect past increases in metabolic needs, meaning that while mitochondria are not producing ATP more intensively in TH-chicks at this time, they retain a higher ATP production capacity. However, to our knowledge, this has not been shown so far and we did not observe a difference in growth trajectories in our study. Alternatively, the harsh environmental conditions, such as the enlarged broods and the husk treatment that all chicks were subjected to in our study, could have limited the TH-treatment to boost growth because of the lack of sufficient nutrients owing to the difficult food availability. It is plausible that while the potential mitochondrial maximal capacity (ETS) to produce ATP was boosted by the treatment, mitochondria were not able to actually produce more ATP at any time point (as suggested by the only marginally elevated OxPhos and CMR values at 14 dph) to support enhanced growth. Overall, growth occurred normally among all individuals, as body mass values at 8 dph (mean, s.d.: 7.54, 1.62) are in line with values reported previously from the same lineage of zebra finches (mean, s.d.: 7.5, 1.7. Sample size = 974) [94]. Nevertheless, difficult food availability is likely to narrow the leeway of growth-enhancing effects without the supply of extra nutrients.

Our study adds to the current state of knowledge regarding the functioning of TH and mitochondria during growth by showing that TH elevates ETS close to fledging. Thus, we have shown that TH enhances aspects of mitochondrial function during development, while the TH-treatment did not affect growth parameters in our study. We assessed the effects of TH on growth in detail by not only considering final and starting values of tarsus length and body mass but also by considering growth curves of body size (tarsus length and body mass) and body condition (SMI) over the course of the treatment. We used GAMMs that allow for varying shapes of curves across groups (e.g. [67,95]), thus offering a powerful tool for assessing treatment effects on avian growth. As a next step for gaining a deeper understanding of the specific modes of action of TH, we suggest that studies consider GH alongside TH, mitochondrial function and growth, and focus on the steepest part of the growth curve when GH is most active [96], ideally alongside different types of environmental conditions.

As expected, our treatment resulted in elevated T3 plasma concentrations within physiological ranges. T3 levels in the TH-chicks were elevated compared with the control group, but as expected from the blood sampling after 1 day, the difference was statistically marginal. It has been shown in Japanese quail (*Coturnix japonica*), that TH would need to be administered twice daily in order to achieve constantly elevated plasma levels [59], presumably because TH is either metabolized directly or decreases as a result of a negative feedback loop in the hypothalamic–pituitary–thyroid axis [92]. We aimed to minimize the handling stress of administration and thus selected a regime of orally dosing chicks every second day during the main growth phase, to achieve regular peaks of elevated T3 levels. To avoid the effect of handling stress on mitochondrial function [58], we sampled the experimental (TH-chicks) and control group one day after administering the last dose of T3. To confirm that our treatment did in fact result in elevated T3 levels shortly after administration, we treated an additional group of chicks (TH-validation, *n* = 7) using the same regime as for the TH-chicks, with the exception of the last dosage. This group was not considered for any other analysis than to confirm the effectiveness of the T3-treatment. The TH-validation group did not receive a dose on 13 dph but was treated on 14 dph, 1 h before blood sampling. As expected, when combining TH-validation and TH-chicks, and comparing them against the control group, we did see a clear elevation in T3 levels. The T3 levels observed in the TH-validation group can be considered elevated within upper physiological ranges when compared with the control group (pmol ml^−1^ TH-validation mean: 2.568, s.e.: 0.313; control mean: 1.68, s.e.: 0.991). We estimated that the peak levels of T3 after administration would occur about 1 h after dosing, by combining the uptake rate reported in humans [60] of around 2.5 h, with the 30–40% faster metabolic rate of birds [61]. We acknowledge that the expected T3 peak after administration could have also occurred before or after the 1 h time point. For this reason, we cannot rule out the possibility that T3 levels briefly exceeded physiological levels in the TH-chicks. However, we are confident that our treatment resulted in elevated levels within physiological ranges since we did not observe any adverse effects on the chicks’ development that would lead us to believe they were hyperthyroidic. Although SMI at 14 dph had the tendency to be lower, growth and development occurred normally, and, importantly, body condition was not meaningfully lower in TH-chicks. In humans, for example, hyperthyroidism is frequently associated with body mass loss and poor body condition [93]. When it comes to mitochondrial function, our aim was to study the effects of elevated TH throughout the main growth phase. T3 plasma levels that are present at the time of sampling may affect mitochondria in a different way than long-lasting adjustments of mitochondrial functioning to the repeated treatment with TH throughout the treatment period [15,19,46]. In different individuals, T3 is probably taken up and metabolized at varying speeds, which may depend for example on the nutritional state at the time of administration and blood sampling. For this reason, we controlled for immediate effects of acute T3 levels on mitochondrial function, by including T3 plasma levels in the statistical models for the mitochondrial traits. We did not find an effect of acute T3 plasma levels on any of the mitochondrial parameters. Nor did any of the results change when T3 levels were removed from the models.

Because our treatment increased T3 levels within physiological levels, a high variability in the resulting T3 concentrations is expected owing to natural variation in endogenous secretion, which is probably the reason for the relatively high variance in our growth and mitochondrial measurements. Furthermore, our simulation of naturalistic challenges in food availability (the addition of husks to the parents’ food dishes) probably increased individual variation in parental care and the food availability for the nestlings [52]. It has been suggested that TH levels are linked to food availability [97] and begging behaviour [34,98], with the latter being adjusted to the parental care an individual receives. This is especially true for large broods (in our study, enlarged broods of six or seven chicks per nest were created at 1 or 2 dph, with a mean brood size of 5.56, s.d.: 0.699 during the chick-rearing period), which increases competition among nestlings for food and space [53]. Taking all these factors into account, it is evident that the interplay of physiological variation, parental care dynamics and brood size (see also [99]) culminates in the observed variances in all our findings. Such complex interactions underscore the importance of investigating TH effects within broader ecological and behavioural contexts.

## 5. Conclusions

The aim of this study was to causally test the role of TH in mediating changes in mitochondria and growth within their natural physiological ranges. While it has been shown in various studies that TH play a crucial role in growth and metabolism, ours is the first to manipulate TH within physiological levels in nestlings during the main growth phase post-hatching to test their effects on both growth and mitochondrial metabolism. In this way, we can show the implications for important fitness-related traits, such as metabolism. We found that elevating TH levels within physiological ranges leads to mitochondrial maximal electron transport chain capacity (ETS), without clearly affecting body mass growth, skeletal growth or body condition. Thus, we can conclude that TH play an important role in shaping mitochondrial structure during development, especially in a challenging environment, which was created through brood enlargement and a food availability challenge in this study. TH secretion has been shown to be adjusted flexibly to environmental influences, and mitochondrial function is also regulated by other factors, such as glucocorticoids. Elevated glucocorticoid concentrations are known to increase in adverse conditions [100] and can cause mitochondrial inefficiency in nestling great tits [58]. Consequently, it is likely that the effects of TH on mitochondrial function are context-dependent, especially during metabolically challenging times such as growth. We suggest future studies to explore how TH modulates mitochondrial function in various environmental conditions, to further our understanding of how individuals adjust their energy metabolism to changing climatic conditions and the consequences for fitness-relevant traits, such as growth rate, fledging size or timing of fledging. Another interesting consideration is that T3 is only one component of a complex growth machinery. One important regulator of bone and muscular growth for example is growth hormone [42]. T3 has permissive effects on growth hormone but does not directly lead to higher growth hormone production itself [101,102]. Thus, growth hormone secretion may be a determining factor in controlling the effects of T3 on growth. We suggest future studies to consider the effects of TH on growth and metabolism alongside further parts of the growth network, such as growth hormone.

## Data Availability

The data and code are available via the following link: [103]. Supplementary material is available online [104].
